# SASDL and RBATQ: Sparse Autoencoder With Swarm Based Deep Learning and Reinforcement Based Q-Learning for EEG Classification

**DOI:** 10.1109/OJEMB.2022.3161837

**Published:** 2022-03-23

**Authors:** Sunil Kumar Prabhakar, Seong-Whan Lee

**Affiliations:** Department of Artificial IntelligenceKorea University 36899 Seoul 02841 South Korea

**Keywords:** Deep learning, EEG, PSO, Q-learning, reinforcement learning

## Abstract

The most vital information about the electrical activities of the brain can be obtained with the help of Electroencephalography (EEG) signals. It is quite a powerful tool to analyze the neural activities of the brain and various neurological disorders like epilepsy, schizophrenia, sleep related disorders, parkinson disease etc. can be investigated well with the help of EEG signals. *Goal*: In this paper, two versatile deep learning methods are proposed for the efficient classification of epilepsy and schizophrenia from EEG datasets. *Methods*: The main advantage of using deep learning when compared to other machine learning algorithms is that it has the capability to accomplish feature engineering on its own. Swarm intelligence is also a highly useful technique to solve a wide range of real-world, complex, and non-linear problems. Therefore, taking advantage of these factors, the first method proposed is a Sparse Autoencoder (SAE) with swarm based deep learning method and it is named as (SASDL) using Particle Swarm Optimization (PSO) technique, Cuckoo Search Optimization (CSO) technique and Bat Algorithm (BA) technique; and the second technique proposed is the Reinforcement Learning based on Bidirectional Long-Short Term Memory (BiLSTM), Attention Mechanism, Tree LSTM and Q learning, and it is named as (RBATQ) technique. *Results and Conclusions*: Both these two novel deep learning techniques are tested on epilepsy and schizophrenia EEG datasets and the results are analyzed comprehensively, and a good classification accuracy of more than 93% is obtained for all the datasets.

## I. Introduction

The physical activities of the nervous system can be comprehensively reflected by the EEG signals [1]. If there is any change in the brain function caused due to neurological disorders, then it can be detected by EEG signals. In the field of medicine, an objective basis for diagnosing certain disorders is provided by the information processing of EEG signals, thereby enabling the clinicians to provide effective treatment for the particular brain disorder. Earlier, a manual detection and analysis of the EEG waveforms was done and due to its intensive labor and long-time consumption, automated classification of EEG signals to diagnose the neurological disorder came into existence [2]. Therefore, classification of EEG signals is quite a vital task with respect to the identification, diagnosis and even prevention of brain related disease. In this paper, the classification of epilepsy EEG signals and schizophrenia EEG signals are dealt in much detail. Epilepsy is a chronic disease characterized by sudden and repeated seizures [3]. Due to various initiating locations and transmission modes of the abnormal electrical activities in brain, different clinical manifestation occurs such as loss of consciousness, limb convulsions, behavioral problems etc [4]. The most prevalent technique to examine the brain activities in epileptic patients is with the help of EEG. For epileptic patients, the EEG signals of their brain activity are split into interictal, pre-ictal and ictal states [5]. An unusual pattern is exhibited in the EEG signals where the seizure occurs. A distributed pattern is also sometimes exhibited in the EEG signal where the seizure occurs. A distinctive pattern is also shown by the EEG signals of interictal state and preictal state. Therefore, to differentiate these epileptic states, these patterns in the EEG signals are highly useful so that the occurrence of a seizure can be known thereby reducing the deadly effects it has on the patients [6]. Seizure detection and classification has been studied for the past two decades with the help of machine learning and deep learning techniques, and a good survey about it can be found in [7], [8] enabling the authors not to repeat the past works again and again. However, the most important ideas incorporating machine learning and deep learning since the past four years is discussed here for the better understanding of the readers. A transfer learning along with semi-supervised learning for seizure classification from EEG signals was proposed by Jiang et al., where the average accuracy was shown to be higher than 95% in most cases [9]. A tunable Q wavelet transform dependent on multiscale entropy was proposed for automated classification of epileptic EEG signals where the highest accuracy of even 100% was achieved in few cases [10]. A local mean decomposition (LMD)-based feature analysis with Support Vector Machine (SVM) was utilized by Zhang and Chen where the classification accuracy reached an accuracy of 98.10% [11]. In the year 2018, for automated classification of epilepsy from EEG signals, deep learning approaches was proposed in [12], [13] and a morphological component analysis based SVM classification was proposed in [14], and these three approaches produced a high classification accuracy of more than 95% as per the consideration of their problem requirement. A scalogram based convolution network from EEG signals was proposed in [15], a matrix determinant-based approach was utilized in [16], cross-bispectrum analysis for seizure detection in [17], a novel random forest model with grid search optimization in [18] are some of the famous works in 2019 and almost all the works have achieved a classification accuracy of 90% to 100% depending on type of case study. In the year 2020, a novel convolutional based neural network model [19], improved Radial Basis Function (RBF) analysis [20], Power Spectral Density (PSD) based deep CNN [21], imagined EEG signal analysis through fully convolutional networks [22], empirical mode decomposition analysis along with its derivative [23], a bat algorithm based SVM [24] are some of the famous works for seizure classification from EEG signals with almost all the works reporting a good classification accuracy of more than 90%. In 2021, a Jacobi polynomial transform based technique with Least Square SVM (LS-SVM) [25], an adaptive synthetic sampling approach [26], a fractal-based seizure detection technique [27], Principal Component Analysis (PCA) based Genetic Algorithm (GA) [28], significance of channel selection techniques [29] are utilized for seizure classification from EEG signals where it reported a classification accuracy of more than 90% for most of the classification cases. In 2022, sparse analysis with deep and transfer learning models were developed with an ensemble cum nature inclined classification for epilepsy classification reporting classification accuracies of more than 90% for epilepsy classification [30].

As the paper discusses schizophrenia classification also from EEG signals, recent literature about it is also discussed in the paper as follows. Schizophrenia is a serious mental disorder where people interpret reality in an abnormal manner Schizophrenia results in a combination of delusion, hallucination, and disordered thinking thereby the daily functions are severely impaired [31]. Therefore, schizophrenia involves a range of problems with cognition, emotion, and behaviour. An exact cause of schizophrenia is not known, but a combination of brain chemistry, genetics and environmental factors may contribute to the development of this disease [32]. EEG signals are a great boon to analyze this disorder and some of the famous works are utilized in this field are as follows. For schizophrenia EEG analysis, the EEG series splitting reported an accuracy of 92.91% [33], deep convolutional neural networks reported 98.07% for non-subject based testing and 81.26% for subject based testing [34], spectral based analysis reporting 96.77% [35], swarm computing techniques with classifiers reporting 92.l7% [31], Short Time Fourier Transform (STFT) with CNN reporting 97.00% [36], Partial Least Squares technique reporting 98.77% [37], multivariate Empirical Mode Decomposition (EMD) reporting 93.00% [38], continuous wavelet transform (CWT) with CNN reporting an accuracy of 98.60% [39], a simple CNN reporting 98.96% [40], sparse depiction with nature inclined classification and deep cum transfer learning reporting 98.72% [30] and Collatz pattern reporting an accuracy of 99.47% [41] are some of the most famous works proposed recently. In this work, the key contributions are as follows and no previous works have been reported in literature using the two developed novel deep learning models.

i) Initially a sparse autoencoder with swarm based deep neural network using PSO was developed for classifying epilepsy and schizophrenia datasets.
ii) Secondly, reinforcement learning based on Q-learning was implemented successfully to classify epilepsy and schizophrenia datasets.

The organization of the work is as follows. Section II explains the development of the SASDL, and Section III explains the RBATQ model. Section IV explains the results and discussion and Section V gives the conclusion.

## II. Development of SASDL Model

An autoencoder model is developed to mitigate the dimensionality of the input [42]. A feedforward neural network is utilized by this form of unsupervised learning and the autoencoder has both encoding and decoding plan An input $x$ is usually trained and $x^{\prime }$ is reconstructed to be quite similar to the input $x$ as much as possible. Many kinds of autoencoders are available in literature such as sparse autoencoder, denoising autoencoder, stacked autoencoder etc [42]. When there is a huge data space, the reconstruction of the raw data by the autoencoder can fail as it might fall into replication of the tasks. The sparse autoencoder has usually lower output dimensions and it persuades the autoencoder in reconstructing the raw data from the most useful features instead of replicating it once again. In this study, a sparse autoencoder is chosen which helps to extract the highly useful patterns which would have a very low dimensionality. These feature vectors are once again selected by the PSO/CSO/BA and finally fed into a simple deep neural network which comprises of two hidden layers along with a Softmax output layer. The input vector to the PSO/CSO/BA is given from the bottleneck of the sparse autoencoder. The bias units are the neurons termed as $(+1)$ and these are added to the feed forward neural network with the help of cost function. In order to get a most preferable reconstruction of the input $x$, this step is highly useful, and it can be achieved without overfitting. The cost function of the autoencoder comprises of three steps. Assuming a dataset with a total of $N$ training samples $(x_{1},x_{2},\ldots,x_{n})$, where the $i^{th}$ input is indicated by $x_{i}$. The reconstruction of the input $x_{i}$ is trained by the developed SAE with the help of function $h_{W,b}(x_{i})$ so that its proximity to $x_{i}$ is very close. The squared error, sparsity term and the weight decay are the three important sections of the cost function. The weight decay aids to avoid overfitting. For all $N$ training samples, the mean square error along with the weight decay and sparsity term is expressed as:

\begin{align*}
J_{sparse} (W,b) &= \frac{1}{N}\sum _{i = 1}^{N} \frac{1}{2}\left\Vert h_{W,b}(x^{i}) - x^{i}\right\Vert\\
&\quad+ \frac{\lambda }{2}\sum _{l = 1}^{n_{l - 1}} \sum _{i = 1}^{s_{l}} \sum _{j = 1}^{s_{l + 1}} (W_{ji}^{l})^{2}\quad + \beta \sum _{j = 1}^{s_{l}}KL(p\Vert \hat{p}_{j}) \tag{1}
\end{align*}

where the sparse penalty term is represented by $\beta$, KL indicates the Kullback–Leibler divergence. The value of $\lambda$ should be carefully chosen because a low value of it leads to overfitting and a high value of $\lambda$ leads to underfitting. Here ReLU is chosen as the activation function represented as $a$, which expresses the average activated value of the hidden layer, and it is represented as:

\begin{equation*}
\hat{p}_{j} = \frac{1}{N}\sum _{i = 1}^{N} (a_{j}^{2}(x^{i})) \tag{2}
\end{equation*}

where $p$ represents the sparsity parameter. The calculation of the sparsity term is usually done to make $\hat{p}_{j}$ look identical and close to $p$ as much as possible. Activation and deactivation of neurons on the hidden layer is done by this parameter.

### A. PSO

A famous population-based algorithm utilized to solve optimization problems is PSO [43]. The number of particles constitute the total population, and each particle indicates a candidate. The best solution is searched for by means of updating the velocity and particle vectors as per the equation:

\begin{align*}
v_{id} (t + 1) &= w \ast v_{id} (t) + c_{1} \ast r_{1} \ast ( P_{id} - x_{id} (t)) + c_{2} \ast r_{2} \\
&\quad \ast (P_{gd} - x_{id} (t)) \tag{3}
\\
x_{id} (t + 1) &= x_{id} (t) + v_{id} (t + 1) \tag{4}
\end{align*}

The velocity of the particle $i$ in the $d^{th}$ dimension is represented as $v_{id}$. The position of the particle $i$ in the $d^{th}$ dimension is represented as $x_{id}$. In the $d^{th}$ dimension, $P_{id}$ represents the local best and $P_{gd}$ represents the global best. The random numbers between 0 and 1 is represented by $r_{1}, r_{2}$. The $w$ represents the inertia weight and $c_{1},c_{2}$ represents the acceleration coefficient for both exploitation and exploration purposes. Due to its versatility as proven in literature, PSO is chosen here in this work and is shown in Algorithm 1.

### B. CSO

When dealing with CSO, the following tree main rules are used in expressing the cuckoo search process [44]. Firstly, one egg is laid by a cuckoo at a particular time and its egg is dropped in a randomly chosen nest. Secondly, only the best nests which possess high quality eggs is progressed and carried on to the next generation. Thirdly, with a fixed number of available host nest, the host birds discover the egg laid by the cuckoo with a probability $p_{a} \in [0,1]$. The host bird can then decide to either eliminate the egg or even abandon the nest completely. The algorithm of the cuckoo search is developed using these three rules and is shown in Algorithm 2. A levy flight is generally implemented when new solutions $x^{(t+1)}$ are generating for a cuckoo ‘$c$’ and is expressed as:

\begin{equation*}
x_{i}^{(t+1)} = x_{i}^{(t)} + \alpha \oplus \mathit{Levy}(\lambda) \tag{5}
\end{equation*}

where $\alpha >0$ denotes the step size. Generally, $\alpha =O(L/10)$ is utilized in most cases, where $L$ denotes the characteristic scale of the problem of interest. For a random walk, the (5) is projected as a stochastic equation. Depending on the current location and the transition probability, the random walk in a Markov Chain is modeled. The entry wise multiplications are expressed by the product $\oplus$. To explore the search space here, the random walk through Levy flight process is more efficient as it has a longer step length. With the help of a Levy distribution, a random walk where random step length is obtained is provided by the Levy flight and is projected as:

\begin{equation*}
\mathit{Levy}\sim u = t^{-\lambda },\ (1< \lambda \leq 3) \tag{6}
\end{equation*}

### C. BA

In this process, for a typical bat algorithm, the following idealized rules are utilized [45]. In order to sense the distance, echo location is used by the bats and the difference between the prey and the different background barriers are known by the bats. In a random manner the bats can fly with a particular velocity $v_{i}$ at a position $x_{i}$. The wavelength of their emitted pulses is adjusted quickly. Based on the proximity of the target, the rate of pulse emission $r\in [0,1]$ can be adjusted. The loudness varies from a large positive value $A_{0}$ to a minimum value $A_{\min }$, though in many ways the loudness can vary. For simplicity purposes, the following approximations can be utilized. Generally, the frequency factor $f$ in a specific range $[f_{\min },f_{\max }]$ correlates to a specific wavelength range $[\lambda _{\min },\lambda _{\max }]$. For an easy implementation, any wavelength can be used depending on the specific problem. By means of adjusting the frequencies the range of the wavelength can be adjusted. While fixing the wavelength $\lambda$, the frequency too can be varied as $\lambda$ and $f$ are closely related. For simplicity reasons, it is assumed that $f\in [0,f_{\max }]$. It is well known that higher frequencies possess short wavelength and can travel only a shorter distance. The typical range is only a few meters for bats and the rate of pulse is in the range of $[0, 1]$, where 1 implies the highest pulse emission rate and 0 implies no pulses and the procedure of it is shown in Algorithm 3.

### D. Overall Framework of the Work

**Fig. 1.**
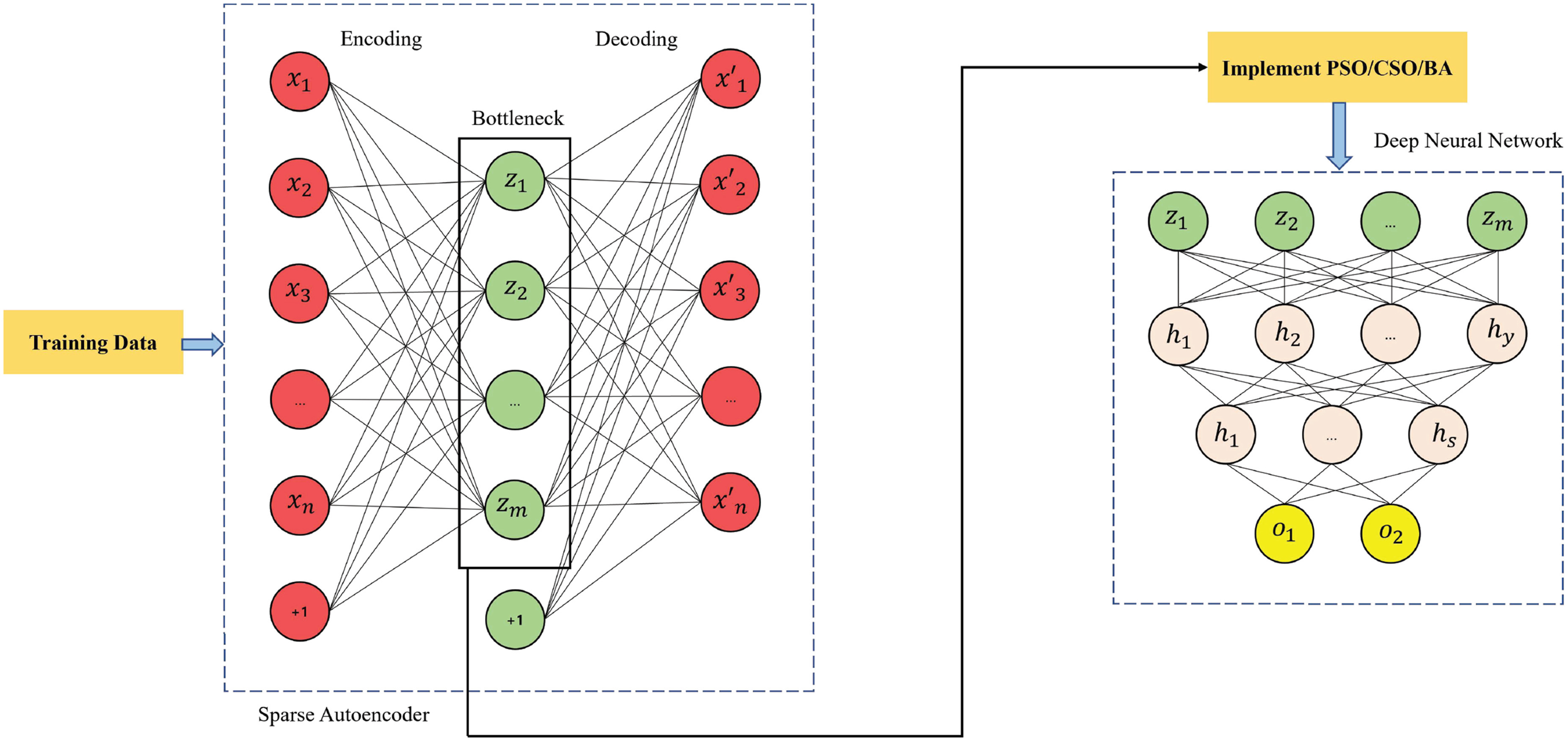
Training of the proposed SASDL method.

**Fig. 2.**
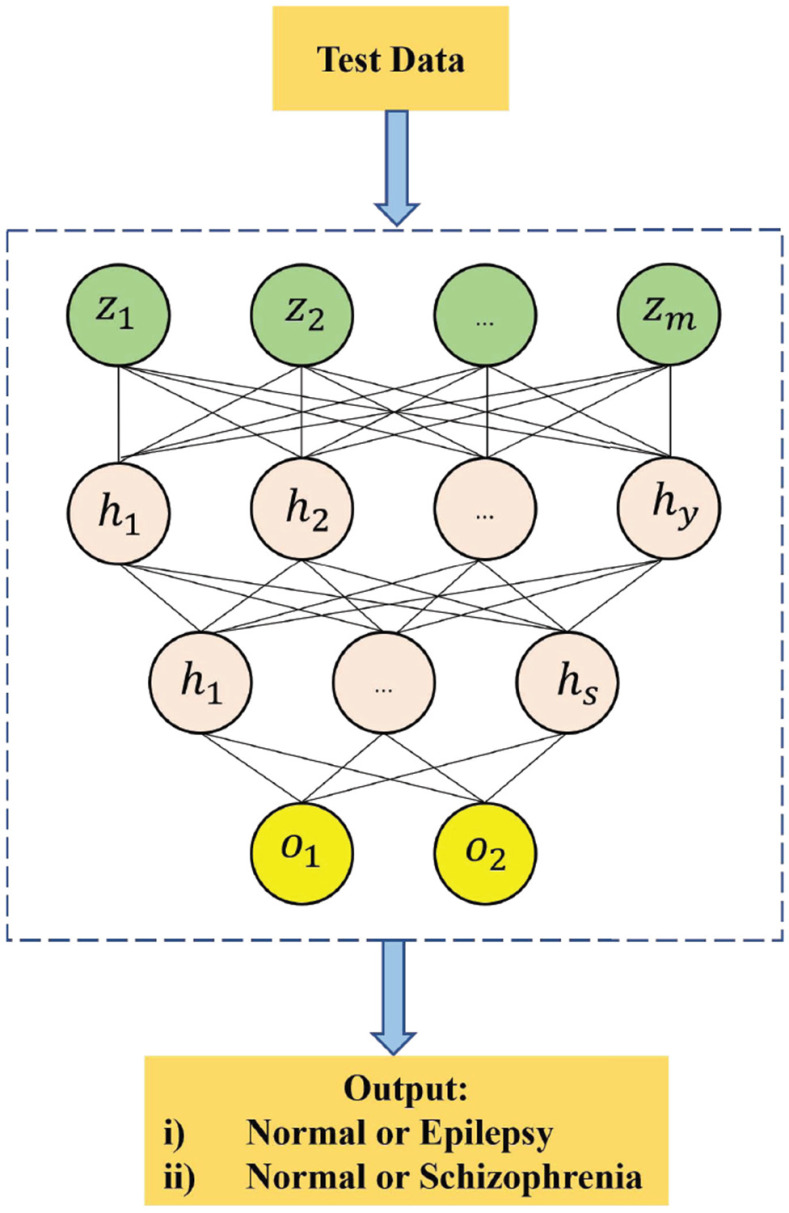
Testing of the proposed SASDL method.

The overall framework with testing and training using the SASDL is depicted from Figs. 1 and 2. Initially, the dataset is split into a training set and test set. The training set is passed to SAE and the bottleneck output of the SAE is fed to the PSO/CSO/BA and then the respective output of it is fed to the DNN. The PSO/CSO/BA is used to select the best particles with the help of Algorithms 1, 2 and 3 respectively.

Algorithm 1: **PSO Implementation to the DNN.**
Input: Population Size $Popul_{size}$, generation *gen*
$popul \leftarrow$ Initialize the particles randomly until the total number of particles reach $Popul_{size}$;

$g_{best,i} \leftarrow Empty,0$

while $i < gen$ do
for particle $p$ in *popul* do
$p \leftarrow$ Position updation of $p$ using standard PSO operation.
*fitness* $\leftarrow$ Compute the fitness for $p$ using the standard fitness evaluation critic
Fitness updation of $p$ by *fitness*
if fitness $>$ fitness of the personal best then
Update the personal best of $p$ with the $p$;
end if
end for
$g_{best} \leftarrow$ Best particle updation among the current $g_{best}$ and *pop*
$i \leftarrow i + 1$;
end while
Return $g_{best}$
Post process it by sending it to the DNN.

As far as the PSO is concerned, the inertia weight is considered as 0.64. The acceleration coefficient $c_{1}$ and $c_{2}$ is considered as 1.524 and the population size is set as 30 and the total number of generations is assigned as 30. All the values were finally chosen after several trial and error-based experimentation efforts.

Algorithm 2: **CSO Implementation to the DNN.**
Input : Objective function $f(x)$, $x=(x_{1},\ldots,x_{d})^{T}$ and the initial population generation with host nests $x_{c}$
while $(t< \mathit{MaxGeneration})$
Random generation of a solution by Levy flight
Evaluation of the fitness $F_{c}$ by the cuckoo
Random choosing of the nest among $n$, say $d$
if $(F_{c}>F_{d})$
New solution replaces $j$
end if
Abandon a fraction $(p_\alpha)$ of worse nests
Generate new nests and its respective solutions
Project only the best or quality solutions
Analyze the current best by ranking the solutions
end while
Post processes it by sending it to the DNN.

As far as the CSO is concerned, to fasten up the local search, the generations of the new solutions by using Levy walk is utilized. The parameters used in our experiments are as follows, nests $n=20$, $\alpha =1.5$ and $p_\alpha =0.45$.

Algorithm 3: **BA Implementation to the DNN.**
Input : Bat population initialization $x_{i}$ and $v_{i}$ $(i=1,2,\ldots,n)$
Frequency initialization $f_{i}$, pulse rate initialization $r_{i}$, loudness initialization $A_{i}$
while ($t$ $< $ Max number of iterations)
Generation of new solutions by frequency adjustment
Velocity updation is done
The location/solutions updation is also done
if $(\mathit{rand}>r_{i})$
Best solution is selected among a set of solutions
Local solution is generated among the best solutions
end if
New solution generation by random flying
if $(\mathit{rand}< A_{i} \mathit{and} f(x_{i})< f(x_{0}))$
Acquire the new solutions
Enhance $r_{i}$ and mitigate $A_{i}$
end if
Ranking of bats to find the current best $x_\ast$
end while
Post process it by sending it to the DNN

As far as BA is concerned, the choosing of the parameters requires some trial-and-error experimentation in this process. By means of randomization, every bat should possess different values of both pulse emission rate and loudness. In this experiment, the initial loudness $A_{i}^{0}$ is considered as 1 and the initial emission rate $r_{i}^{0}$ is chosen as 0.5 as the values can be between $r_{i}^{0}\in [0,1]$. If there is an improvement in the new solution, there will naturally be an updation of the loudness and emission rates, implying that the bats are progressing towards reaching the optimal solution. In our experiment, the value of $n$ is chosen as 40 virtual bats.

## III. Development of RBATQ Model

To analyze the decision process of reinforcement learning, in this paper, three deep learning techniques are utilized such as Bidirectional LSTM, attention mechanism along with Tree LSTM. To get the control policy, Q-learning algorithm is utilized here in this work.

### A. Reinforcement Learning (RL)

In order to learn the control policies of the agent in an efficient manner, the most commonly used framework is RL, and it is done by means of active interaction with its environment [46].

*State:* Three states such as the initial state $s_{1}$, transition state $s_{2}$ along with the end state $s_{e}$ is present in the internal state $S$ of the environment. Representing the state directly from the signal is quite a difficult task as there are no appropriate measures to assess it and therefore to extract the features of signal, deep learning techniques are used which helps to indicate the circumstances in the decision process. Initially, to realize the feature extraction, a bidirectional LSTM [47] is used and to generate the initial state, attention-based methods [48] are used and it is represented as $s_{1} = Att (X;\theta _{1})$. To create the transition state $s_{2}$, Tree-LSTM [49] is utilized here, and it is represented as $s_{2} = Tree(X;\theta _{2})$. $X$ indicates the features of the input signal and the state parameters are expressed by $\theta _{1}$ and $\theta _{2}$ respectively.

*Action:* In the environment, there are quite a collection of predefined actions denoted by $A$, such as action $a_{1}$, action $a_{2}$, action $a_{3}$ respectively. The initial decision decides to consider $a_{1}$ or $a_{2}$, and the next decision decides to consider $a_{3}$ or $a_{4}$. For every action, the reward obtained is represented by $R = r_{1},r_{2},r_{3},r_{4}$. In a state $S$, an action ‘$a$’ is usually considered by the agent and a reward ‘$r$’ is received from the environment. The transition adaption of the decision procedure is chosen accordingly.

*Transition and Reward function:* The agent considering $a_{1}$ at $s_{1}$ is then transmitted to $s_{e}$ by means of effectively utilizing a state transition tuple $(s_{1},a_{1}, r_{1}, s_{e})$. An agent usually receives a reward $r_{1}$ if the judgement of $a_{1}$ is correct. If the judgement of $a_{1}$ is incorrect, then in order to push the utilizing judgement of the initial decision, $r_{1}$ can be set accordingly. The rest of the state transition tuples and its respective reward function can be assigned in a similar manner.

### B. BiLSTM Layer

Every LSTM component in the BiLSTM layer comprises of three multiplicative gates such as input gate $i_{t}$, forget gate $f_{t}$ and output gate $o_{t}$. The proportion of information can be controlled by these gates and helps it to progress on to the next time step. In each LSTM unit, a memory cell $c_{t}$ is also kept which helps to analyze the preceding state thereby the features of the current input signal can be well memorized. For every LSTM unit, the data sources are as follows: the feature vector $x_{t}$ at time $t$, hidden state vector $h_{t - 1}$ and $h_{t + 1}$ (before and after time $t$, along with the cell vector $c_{t - 1}$). The implementation of forward passes are as follows:

\begin{align*}
i_{t} &= \sigma ({W_{xi} x_{t} + W_{hi} h_{t - 1} + W_{ci} c_{t - 1} + b_{i}})\tag{7}
\\
f_{t} &= \sigma ({W_{xf} x_{t} + W_{hf} h_{t - 1} + W_{cf} c_{t - 1} + b_{f}}) \tag{8}
\\
g_{t} &= \tanh ({W_{xc} x_{t} + W_{hc} h_{t - 1} + W_{cc} c_{t - 1} + b_{c}}) \tag{9}
\\
c_{t} &= i_{t} g_{t} + f_{t} c_{t - 1}\tag{10}
\\
o_{t} &= \sigma ({W_{xo} x_{t} + W_{ho} h_{t - 1} + W_{co} c_{t} + b_{o}}) \tag{11}
\\
h_{t} &= o_{t} \tanh ({c_{t}}) \tag{12}
\end{align*}

where the weight matrices are represented by $W$, bias vectors are represented as $b$. The subscripts indicate the meaning as per the name suggestion as it is commonly represented in BiLSTM concept [47]. The logistic function is indicated by $\sigma$. The execution of the backward passes with respect to time are carried out in a same fashion as the forward passes. At a time $t$, the hidden state vectors of two directions $h_{t}$ and $h_{t}^{\prime }$ are computed simultaneously in the BiLSTM layer, therefore past features and future features can be efficiently utilized in a specific time frame. The hidden state vectors of two directions $h_{t}$ and $h_{t}^{\prime }$ is passed to a Softmax layer at a particular time $t$ and it is represented as:

\begin{equation*}
y_{t} = \text{soft }\max (W_{hy}h_{t} + W_{h^{\prime }y}h_{t}^{\prime } + b_{y}) \tag{13}
\end{equation*}

Here the weight matrices are expressed by $W$ and the bias vector is represented by $b$. Attention mechanism is applied to the BiLSTM.

### C. Tree LSTM

This concept was implemented in the field of natural language processing (NLP) however, the idea has been tried to biosignal processing for the first time in this paper. The development of the tree LSTM starts from its leaf node, and it is done in a recursive manner up to the root [49]. On the hidden state vector of the antecedent element, the non-linear transformation is carried out so that $s_{2}$ is generated. It serves as an important transition predicament in the decision process and therefore $s_{2}$ is denoted as: $s_{2} = Tree(X;\theta _{2})$ where $\theta _{2}$ indicates all the essential criterion in the Tree-LSTM. Once the transition state $s_{2}$ is generated, it is progressed to a Softmax output layer so that $y_{r}$ is obtained which indicates the probability of various kinds for a relation mention. A category with the highest probability is chosen, so that $a_{3}$ or $a_{4}$ can be determined easily.

\begin{equation*}
y_{r} = soft\max ({W_{sy} s_{2} + b_{y}}) \tag{14}
\end{equation*}

Here the weight metric is represented by $W$ and the bias vector is specified by $b$. A softmax layer is utilized at every dependency tree so that the category for the root node is predicted when the given inputs $X$ are discovered at its respective children nodes.

### D. Q-Learning

An approved form of reinforcement learning technique is Q-learning algorithm [50]. For the agent, an optimal state-action value function $Q(s,a)$ can be easily used to learn it. By means of consultation of $Q(s,a)$, the agent considers an action $a$ in state $s$, which is nothing but the simple estimation of the action’s anticipated long-term reward. By means of analyzing a sequence of actions, some cumulative rewards can be maximized. For every state-action pairs, it is quite difficult to obtain $Q(s,a)$ as the state space is infinite in the decision process. Therefore, using a novel network, $Q(s,a)$ is approximated which can specify $Q(s,a)$ as a parameterized outcome represented as $Q_{\eta } (s,a) = MLP ({\varphi ({X;\theta }),a,\eta })$. $s_{1} = Att ({X;\theta _{1}})$ is referred by $s_{1} = \varphi ({X;\theta _{1}})$ and $s_{2} = Tree ({X;\theta _{2}})$, where $\theta$ is calculated by means of pretraining the deep learning models. The parameter in the neural network is represented by $\eta$ and it is learnt by implementing the famous stochastic gradient descent step with the help of RMSprop. The degree of approximation is measured with respect to the least squares error in order to estimate the real value function $Q^{\pi }$ as follows:

\begin{equation*}
E_{\eta } = E{[ { ({Q_{\pi } ( {s,a}) - Q_{\eta } ({s,a}) })^{2}} ]} \tag{15}
\end{equation*}

where $E$ represents the least square value. Instead of the real value function $Q^{\pi } (s,a)$, the estimated value function $Q_{\eta } (s,a)$ is used by the Q-learning. In the middle of the estimation $Q_{\eta } (s,a)$ and the expectation $Q^{\pi } (s,a)$, the discrepancy is reduced when the parameters are updated during every epoch. There is a continuous updation of values when the agent progresses from a random $Q_{\eta } (s,a)$ by means of utilizing the decisions and obtains the suitable reward. By carefully selecting the actions with the highest $Q_{\eta } (s,a^{\prime \prime })$, the agent can expand its future rewards accordingly. Ultimately, the control policy $\pi$ is obtained by the Q-learning algorithm. When the training procedure is carried out, BiLSTM, attention layer along with Tree-LSTM are pre-trained initially. All the parameters in BiLSTM are indicated as $\theta _{0}$, all the parameters in attention layer are specified as $\theta _{1}$ and all the parameters in Tree-LSTM is indicated as $\theta _{2}$ are these are the main training parameters used. Deep learning is used to represent the features and RL is used to combine these three tasks in the final decision process. The standard conventional pipeline architectures fail to enable the information to flow in a sequential manner, but this RL method combines all the tasks in a sequential manner and allows to make decisions too. The decisions may have problems initially but after several epochs, a good stability can be obtained. A global updation of the parameters $s$ is done in this architecture and therefore an eventual convergence is achieved later. Hence the feedback from decision-making can be obtained easily by the RL method thereby enabling the data to progress easily in the global architecture. The Q-learning training procedure is expressed in Algorithm 4.

Algorithm 4: **Q-Learning Training Procedure for the Proposed RBATQ Method.**
Start BiLSTM, Attention mechanism and Tree-LSTM with random parameters

$\eta = 0$

Pre-training of BiLSTM, Attention mechanism and Tree-LSTM process
For every epoch $=$ 1,2 do
For every input signal $X$ do
Utilize deep learning model for automated feature extraction of $X$ and produce $S_{1}$ and $S_{2}$
For t $=$ 1,2 do
$r,s^{\prime } = \text {reward}$ and state after considering the action $\pi (s)$
  \begin{equation*}
a\prime = \pi ({s\prime })
\end{equation*}
Implement Gradient descent step:
  \begin{align*}
- {\frac{{\partial E_{\eta } } }{{\partial \eta } }} &= E{\left[ {2( {Q_{\pi } ({s,a}) - Q_{\eta } ({s,a}) }){\frac{{\partial Q_{\eta } (s,a)}}{{\partial \eta } }}} \right]}\\
Q_{\pi } (s,a) &= {\frac{{1}}{{t}}}r + {\frac{{t - 1}}{{t}}}Q^{\pi } ({s,^{\prime }a^{\prime }})
\end{align*}
Updation Process:
  \begin{equation*}
\eta = \eta + \alpha \left({{\frac{{1}}{{t}}}r + {\frac{{t - 1}}{{t}}}Q_{n} ({s,^{\prime }a^{\prime }}) - Q_{\eta } (s,a){\frac{{\partial Q_{\eta } (s,a)}}{{\partial \eta } }}} \right)
\end{equation*}
$\alpha = \text {update}$ step, $r = \text {reward}$ function
$ ({s,^{\prime }a^{\prime }}) = \text {state}$ action pair

$\pi (s) = {\mathop {\arg \max } _{a^{\prime \prime }}}\ Q_{\eta } ({s,a^{\prime \prime }})$

$s = s,^{\prime }a = a^{\prime }$

End for
End for
End for

## IV. Results and Discussion

The proposed deep learning models has been initially evaluated on the University of Bonn dataset where it deals with epilepsy classification. Then the proposed deep learning models has been evaluated on the dataset obtained for Institute of Psychiatry and Neurology, Poland. As far as the Bonn dataset is concerned, the epilepsy datasets are categorized into A, B, C, D and E sets. The normal category dataset is present in set A and set B, the interictal category dataset is present in set C and set D and the ictal category dataset is present in set E. The classification problems discussed here are A-E, B-E, C-E, D-E, AB-E, AC-E, CD-E, ACD-E, ABCD-E. As far as the schizophrenia dataset is considered, it is just normal case versus schizophrenia case. All the explicit datasets for it are given in the reference [51], [52]. In the epilepsy dataset, 100 single channel EEG recordings are present which has a sampling rate of 173.61 Hz along with a time duration of 23.6 seconds. The sampling of these time series is done into 4097 data points and then all these 4097 data points are further split into 23 chunks, here about 2300 samples are present in each category. For the deep learning techniques, the 2300 EEG signals are randomly divided into ten non-overlapping folds due to the adoption of a 10-fold cross validation technique utilized here for evaluation. When dealing with schizophrenia datasets, each channel has about 225,000 samples and therefore the data is specified into a matrix format of $[5000 \times 45]$. As it has about 19 channels, it is specified exactly as $[5000 \times 45 \times 19]$. When the implementation of deep learning techniques happens, the schizophrenia EEG samples are randomly divided into ten non-overlapping folds due to the adoption of a 10-fold cross validation technique here. The dimensionality representation of the input is reduced by SAE where the size of the input is about $(4097 \times 100)$ for epileptic dataset and $(5000 \times 45)$ for schizophrenia dataset. It is reduced to about $(2500 \times 50)$ for epileptic dataset and $(5000 \times 15)$ for schizophrenia dataset. These useful features are provided by the bottleneck of the SAE that is fed to the PSO/CSO/BA. The size of the bottleneck comprises of 9000 hidden units. After it is passed to PSO/CSO/BA, a total of 4500 features are obtained. The classifier comprises of two hidden layers and an output layer where the sizes of units are expressed as 2250, 500 and 2 respectively. To specify the probability of each class, Softmax regression is utilized in the output layer. In between the fully connected neural networks, dropout is utilized to prevent the overfitting. In between the two classes, the maximum probability is chosen as the final decision of the classifier. To compute the cost function of the classifier, cross entropy is utilized and then a weight decay term was added to it subsequently. The cost function is minimized by the SAE and the PSO/CSO/BA selects the most important features as it is given as input to the DNN. The completion of the training process is done in about 50 iterations and the batch size was set as 10. The value of the sparsity parameter $p$ is chosen as 0.08, the weight decay $\lambda$ is set as 0.01 and the sparse penalty term $\beta$ was chosen as 4 respectively. To adjust the classifier parameters, fine tuning of the deep neural network classifier was done on the last 20 iterations so that the cost function of the Softmax was minimized. For parameter updation, Adam optimizer is used. The evaluation of the model was done using a 10-fold cross validation technique. As for the RBATQ deep learning model, the hyperparameters implemented are as follows. The state size for all the LSTM units is set as 250 and the dimension of the hidden layer is fixed as 100. The non-linear function utilized is tanh. The dropout rate is set as 0.75, initial learning rate is 0.002. Mini batch size is set as 25 and the constraint of maximum norm regularization is set as 3. The performance metrics analyzed here are sensitivity, specificity, and accuracy and is tabulated in Table I.

On analyzing Table I, it is inferred that for the epileptic dataset (A-E), a good classification accuracy of 97.75% is obtained when utilizing RBATQ model, 98.55% accuracy with SASDL-PSO model, 98.50% accuracy with SASDL-CSO model, 98.26% accuracy with SASDL-BA model and 96.52% for SAE with DNN model. For the epileptic dataset (B-E), a good classification accuracy of 97.42% is obtained when utilizing RBATQ model, 97.85% accuracy with SASDL-PSO model, 97.64% accuracy with SASDL-CSO model, 97.55% accuracy with SASDL-BA model and 98.03% for SAE with DNN model. For the epileptic dataset (C-E), a good classification accuracy of 94.57% is obtained when utilizing RBATQ model, 98.11% accuracy with SASDL-PSO model, 96.62% accuracy with SASDL-CSO model, 96.18% accuracy with SASDL-BA model and 95.8% for SAE with DNN model. For the epileptic dataset (D-E), a good classification accuracy of 94.74% is obtained when utilizing RBATQ model, 97.52% accuracy with SASDL-PSO model, 96.56% accuracy with SASDL-CSO model, 96.74% accuracy with SASDL-BA model and 95.91% for SAE with DNN model. For the epileptic dataset (AB-E), a good classification accuracy of 94.4% is obtained when utilizing RBATQ model, 98.4% accuracy with SASDL-PSO model, 98.04% accuracy with SASDL-CSO model, 97.96% accuracy with SASDL-BA model and 98% for SAE with DNN model. For the epileptic dataset (CD-E), a good classification accuracy of 94.29% is obtained when utilizing RBATQ model, 98.55% accuracy with SASDL-PSO model, 96.57% accuracy with SASDL-CSO model, 95.46% accuracy with SASDL-BA model and 95.22% for SAE with DNN model. For the epileptic dataset (AC-E), a good classification accuracy of 94.4% is obtained when utilizing RBATQ model, 97.25% accuracy with SASDL-PSO model, 95.90% accuracy with SASDL-CSO model, 95.51% accuracy with SASDL-BA model and 95.49% for SAE with DNN model. For the epileptic dataset (ACD-E), a good classification accuracy of 93.89% is obtained when utilizing RBATQ model, 97.89% accuracy with SASDL-PSO model, 96.63% accuracy with SASDL-CSO model, 95.87% accuracy with SASDL-BA model and 95.45% for SAE with DNN model. For the epileptic dataset (ABCD-E), a good classification accuracy of 94.04% is obtained when utilizing RBATQ model, 98.48% accuracy with SASDL-PSO model, 96.99% accuracy with SASDL-CSO model, 95.95% accuracy with SASDL-BA model and 95.64 % for SAE with DNN model. For the epileptic dataset (BCD-E), a good classification accuracy of 93.68% is obtained when utilizing RBATQ model, 98.07% accuracy with SASDL-PSO model, 97.46% accuracy with SASDL-CSO model, 97.21% accuracy with SASDL-BA model and 97.6% for SAE with DNN model. For the schizophrenia dataset, a good classification accuracy of 94.97% is obtained when utilizing RBATQ model, 97.95% accuracy with SASDL-PSO model, 96.22% accuracy with SASDL-CSO model, 96% accuracy with SASDL-BA model and 96% for SAE with DNN model.

**TABLE I:** Performance Analysis of the Proposed Deep Learning Techniques

Performance Metrics (%)	Datasets	Data Learning Models				
		SAE	Proposed	Proposed	Proposed	Proposed
		with	SASDL	SASDL	SASDL	RBATQ
		DNN	Model - PSO	Model - CSO	Model - BA	Model
Sensitivity	Epileptic Dataset (A-E)	97.01	98.99	98.89	98.21	97.21
	Epileptic Dataset (B-E)	98.11	98.02	98.00	97.11	96.74
	Epileptic Dataset (C-E)	96.30	98.34	97.13	96.34	94.16
	Epileptic Dataset (D-E)	95.34	96.47	95.99	95.98	93.49
	Epileptic Dataset (AB-E)	97.89	98.69	98.02	97.91	93.79
	Epileptic Dataset (CD-E)	95.34	98.19	96.12	95.49	92.11
	Epileptic Dataset (AC-E)	94.87	96.79	94.99	94.89	94.79
	Epileptic Dataset (ACD-E)	94.79	97.78	96.16	94.91	92.90
	Epileptic Dataset (ABCD-E)	95.10	98.24	96.87	95.12	94.99
	Epileptic Dataset (BCD-E)	97.10	98.16	97.32	97.31	93.35
	Schizophrenia Dataset	94.89	97.89	95.18	95.01	94.93
Specificity	Epileptic Dataset (A-E)	96.04	98.12	98.12	98.31	98.29
	Epileptic Dataset (B-E)	97.96	97.69	97.28	97.99	98.11
	Epileptic Dataset (C-E)	95.31	97.89	96.11	96.03	94.99
	Epileptic Dataset (D-E)	96.48	98.58	97.13	97.51	95.99
	Epileptic Dataset (AB-E)	98.12	98.11	98.07	98.01	95.01
	Epileptic Dataset (CD-E)	95.11	98.92	97.03	95.44	96.47
	Epileptic Dataset (AC-E)	96.11	97.71	96.82	96.14	94.01
	Epileptic Dataset (ACD-E)	96.12	98.01	97.11	96.83	94.89
	Epileptic Dataset (ABCD-E)	96.18	98.73	97.12	96.78	93.09
	Epileptic Dataset (BCD-E)	98.10	97.99	97.61	97.12	94.02
	Schizophrenia Dataset	97.11	98.01	97.27	96.99	95.02
Classification Accuracy	Epileptic Dataset (A-E)	96.525	98.555	98.505	98.26	97.75
	Epileptic Dataset (B-E)	98.035	97.855	97.64	97.55	97.42
	Epileptic Dataset (C-E)	95.805	98.115	96.62	96.185	94.57
	Epileptic Dataset (D-E)	95.91	97.525	96.56	96.745	94.74
	Epileptic Dataset (AB-E)	98.005	98.4	98.045	97.96	94.40
	Epileptic Dataset (CD-E)	95.225	98.555	96.575	95.465	94.29
	Epileptic Dataset (AC-E)	95.49	97.25	95.905	95.515	94.40
	Epileptic Dataset (ACD-E)	95.455	97.895	96.635	95.87	93.89
	Epileptic Dataset (ABCD-E)	95.64	98.485	96.995	95.95	94.04
	Epileptic Dataset (BCD-E)	97.6	98.075	97.465	97.215	93.68
	Schizophrenia Dataset	96	97.95	96.225	96	94.97

**Fig. 3.**
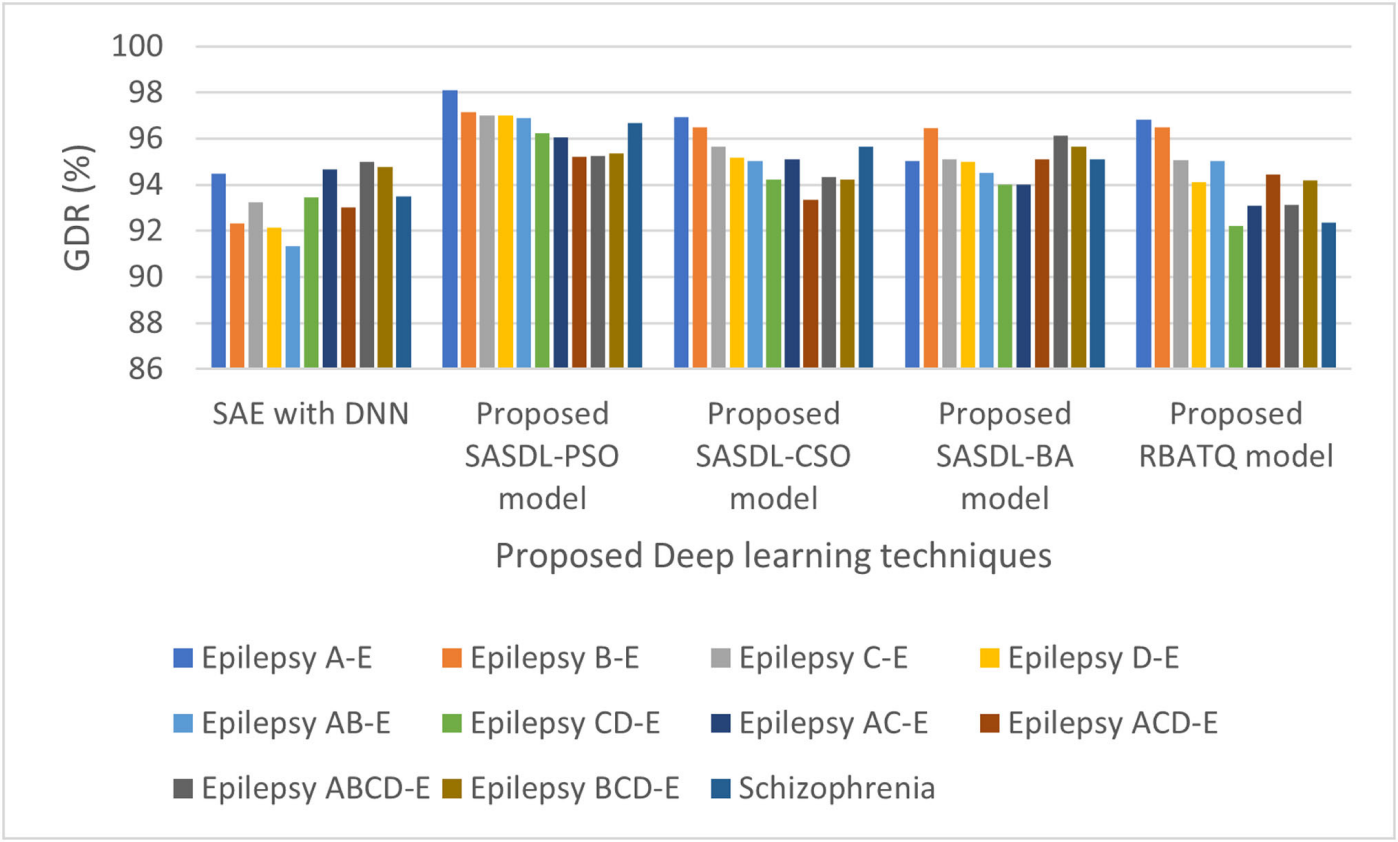
Performance analysis of Good Detection Rate (GDR) %.

**Fig. 4.**
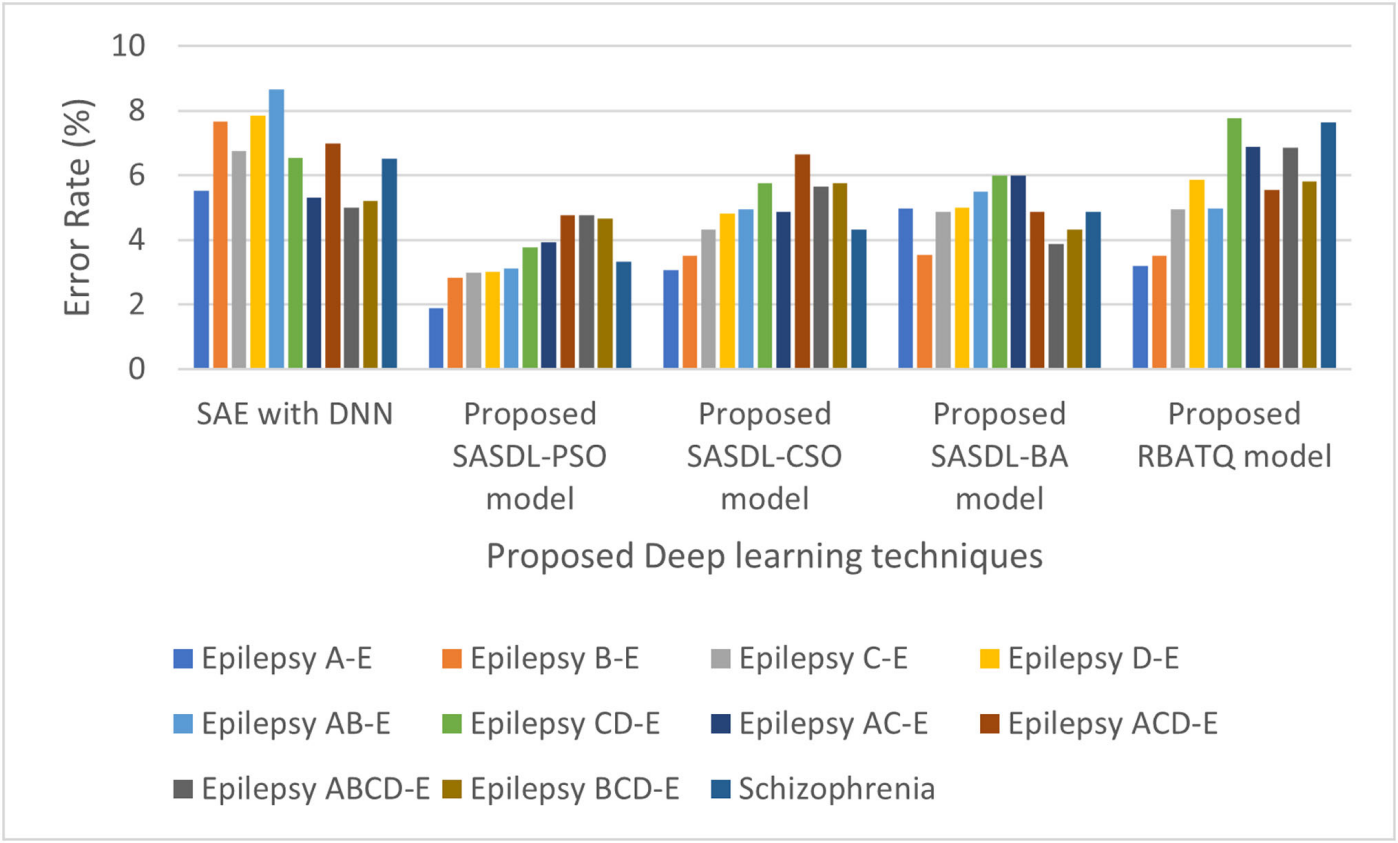
Performance analysis of error rate (%).

The Good Detection Rate (GDR) and Error Rate Analysis for the Deep learning models is plotted in Figs. 3 and 4 respectively. As inferred from Fig. 3 for the proposed SASDL-PSO model produces a high GDR and then it is followed by the proposed SASDL-CSO model and the SASDL-BA model. The proposed RBATQ model and the ordinary SAE-DNN model produce a comparatively low GDR when compared to the other classifiers. As inferred from Fig. 4, a low error rate is obtained for the proposed SASDL-PSO model. A high error rate is obtained for the SAE-DNN model, and the remaining three models too have a slightly higher error rate than the proposed SASDL-PSO model.

### A. Comparison With Previous Works for Epilepsy Bonn Dataset and Schizophrenia Dataset

Though thousands of papers are published online every year in epilepsy and schizophrenia classification, a few selected important and recent works which have analyzed many combinations of the epilepsy problem has been considered and the results have been compared with them and reported in Tables II and III. The best result of 98.55% has been obtained for the A-E problem with the proposed SASDL-PSO model, 98.03% for B-E problem with the SAE-DNN model, 98.11% for the C-E problem with the proposed SASDL-PSO model, 97.52% for D-E problem with the proposed SASDL-PSO model, 98.4% for AB-E problem with the proposed SASDL-PSO model, 98.55% for CD-E problem with the proposed SASDL-PSO model, 97.25% for AC-E problem with the proposed SASDL-PSO model, 97.89% for ACD-E problem with the proposed SASDL-PSO model, 98.48% for ABCD-E problem with the proposed SASDL-PSO model, and 98.07% for BCD-E problem with the proposed SASDL-PSO model. For schizophrenia classification, the best result of 97.95% is obtained with the proposed SASDL-PSO model. The proposed results have more or less reached the similar results when compared to the previous state of the art results, sometimes giving more classification accuracy than the previous results and sometimes giving less classification accuracy than the previous results by a minor margin. The main intention of this work is to analyze a swarm based deep neural networks along with a Reinforcement based Q-learning for epilepsy and schizophrenia datasets and the results are projected.

**TABLE II:** Comparison With Previous Results for the Epilepsy Bonn Dataset

**Reference and Work done**	**Year**	**A-E**	**B-E**	**C-E**	**D-E**	**AB-E**	**CD-E**	**AC-E**	**ACD-E**	**ABCD-E**	**BCD-E**
[10] - Tunable Q wavelet	2017	100	100	99.50	98	–	–	–	–	99	–
transform analysis											
[11] - LMD based features	2017	100	–	–	98.10	–	–	–	–	–	–
with SVM											
[12] - Automated analysis using	2018	100	99.6	99.1	99.4	99.8	99.7	–	–	99.7	–
deep learning											
[15] - Scalogram based CNN	2019	99.50	99.50	98.50	98.50	–	–	–	–	–	–
[16] - Matrix determinant	2019	99.45	96.06	97.60	97.60	97.10	96.85	96.50	96.00	97.20	–
analysis design											
[19] - End-to-End deep neural	2020	99.52	99.11	98.02	97.63	99.38	98.03	–	–	98.76	–
network model											
[30] - Ensemble and nature inclined	2022	98.15	98.16	97.89	95.34	97.84	–	–	–	–	–
classification with sparse modelling											
cum deep learning											
SAE with DNN		96.52	98.03	95.80	95.91	98.00	95.22	95.49	95.45	95.64	97.6
Proposed SASDL - PSO model		98.55	97.85	98.11	97.52	98.4	98.55	97.25	97.89	98.48	98.07
Proposed SASDL - CSO model		98.50	97.64	96.62	96.56	98.04	96.57	95.90	96.63	96.99	97.46
Proposed SASDL - BA model		98.26	97.55	96.18	96.74	97.96	95.46	95.51	95.87	95.95	97.21
Proposed RBATQ		97.75	97.42	94.57	94.74	94.4	94.29	94.4	93.89	94.04	93.68

**TABLE III:** Comparison With Previous Results for the Schizophrenia Dataset

**Reference and Work done**	**Year**	**Accuracy (%)**
[34] – Deep CNN	2019	81.26 – subject
		based testing
		98.07 – non-subject
		based testing
[33] – Non linear signal processing methods	2019	92.91
[36] – Multivariate EMD analysis	2020	93.00
[39] – Transfer learning with deep CNN	2020	98.60
[31] – Swarm intelligence computing	2020	92.17
techniques		
[40] – Spectral features with CNN	2020	98.96
[37] – Nature inspired optimization	2020	98.77
techniques		
[41] – Collatz pattern analysis technique	2021	99.47
[30] – Ensemble and nature inclined	2022	98.72
classification with sparse modelling		
cum deep learning		
SAE with DNN		96
Proposed SASDL – PSO model		97.95
Proposed SASDL – CSO model		96.22
Proposed SASDL – BA model		96
Proposed RBATQ		94.97

## V. Conclusion

To study and analyze the neuronal dynamics within the human brain, the most standard tool utilized by the researchers and clinicians is EEG. For the EEG dependent analysis of various neurological disorders, visual inspection of these huge datasets is very difficult. Therefore, feature extraction techniques and automated classification schemes have been developed in the past. With the advent of deep learning, manual feature extraction is not necessary as it is aided by the deep learning process itself. In this paper, two novel deep learning techniques one with the help of swarm intelligence and another with the help of Reinforcement learning such as SASDL and RBATQ are proposed in this paper and tested for two EEG datasets such as epilepsy dataset and schizophrenia dataset. The highest classification accuracy of 98.55% was obtained with the proposed SASDL-PSO method and 97.75% was obtained with the proposed RBATQ method for epilepsy dataset. The highest classification accuracy of 97.95% was obtained with the proposed SASDL-PSO method and 94.97% was obtained with the proposed RBATQ method for schizophrenia dataset. Future works aim to develop more interesting deep learning models to classify the EEG datasets with a high classification accuracy. Moreover, these developed deep learning models are planned to be implemented for other biosignal datasets such as Electrocardiogram (ECG), Photoplethysmogram (PPG), Electrooculogram (EOG) etc for the diagnosis of various medical disorders.
